# To burn or not to burn: governance of wildfires in Australia

**DOI:** 10.5751/es-14801-290108

**Published:** 2024-01-01

**Authors:** Sarah Clement, Ahjond Garmestani, Jo Ann Beckwith, Pele J. Cannon

**Affiliations:** 1 Fenner School of Environment and Society, College of Science, Australian National University,; 2 School of Social Sciences, University of Western Australia,; 3 Department of Geography and Planning, School of Environmental Sciences, University of Liverpool,; 4 U.S. Environmental Protection Agency, Office of Research and Development,; 5 Utrecht Centre for Water, Oceans and Sustainability Law, Utrecht University,; 6 Department of Environmental Sciences, Emory University,; 7 Center for Resilience in Agricultural Working Landscapes, School of Natural Resources, University of Nebraska-Lincoln,; 8 Independent Researcher

**Keywords:** adaptive governance, bushfires, natural hazards, prescribed burning, regional planning, risk mitigation, transformation, wildfire governance

## Abstract

Globally, wildfires are increasing in extent, frequency, and severity. Although global climate change is a major driver and large-scale governance interventions are essential, focusing on governance at smaller scales is of great importance for fostering resilience to wildfires. Inherent tensions in managing wildfire risk are evident at such scales, as objectives and mandates may conflict, and trade-offs and impacts vary across ecosystems and communities. Our study feeds into debates about how to manage wildfire risk to life and property in a way that does not undermine biodiversity and amenity values in social-ecological systems. Here, we describe a case study where features of adaptive governance emerged organically from a dedicated planning process for wildfire governance in Australia. We found that a governance process that is context specific, allows for dialogue about risk, benefits, and trade-offs, and allows for responsibility and risk to be distributed amongst many different actors, can provide the conditions needed to break down rigidity traps that constrain adaptation. The process enabled actors to question whether the default risk management option (in this case, prescribed burning) is aligned with place-based risks and values so they could make an informed choice, built from their participation in the governance process. Ultimately, the community supported a move away from prescribed burning in favor of other wildfire risk management strategies. We found that the emergent governance system has many features of adaptive governance, even though higher level governance has remained resistant to change. Our study offers positive insights for other governments around the world interested in pursuing alternative strategies to confronting wildfire risk.

## INTRODUCTION

In recent years, fire-prone areas have experienced a significant increase in the frequency, intensity, and severity of wildfires. Nowhere has this pattern been more noticeable than in Australia, where worsening fire conditions and expansion of the rural-urban interface (RUI) have resulted in increased exposure of communities to more severe wildfires.^[1]^ Although climate change is known to be a key driver of increasingly dangerous fire conditions (Canadell et al. 2021), mitigating or adapting to anticipated climate change impacts is not the primary focus of efforts to mitigate the risks of wildfires. A strong focus in the political realm remains on how changing governance might lead to better outcomes for people and nature, as is evident in the dozens of state and federal inquiries of major Australian wildfire events. These inquiries have produced > 1300 recommendations since 2009 alone (Bushfire & Natural Hazards CRC 2020), many of which devolve responsibility for risk mitigation to communities and local governments (Akter and Grafton 2021). A wildfire requires fuel, and that fuel comes in the form of vegetation. Governance and policy reforms have focused on “enhanced” prescribed burning programs to reduce fuel loads.

Consequently, Australia’s environmental and land management agencies have been under increasing pressure to burn more hectares and meet substantial burning targets, focusing wildfire risk management even further on prescribed burning. Although the intention of such policies (in encouraging large-scale burning) is to make communities safer, in recent years these practices have sparked a series of debates and controversies around whether prescribed burning (as currently practiced) reduces risk to life and property while at the same time increasing vulnerability or reducing resilience over the long term (Clement 2021). While scientists continue to debate these issues and explore how wildfire risk management needs to vary across different ecosystems (c.f. Clarke et al. 2022, Zylstra et al. 2022), this nuance is not always captured in how fire-prone landscapes are governed or managed in practice. Historical policies and practices also create path dependencies and even rigidity traps, where the status quo is maintained even when it is maladaptive (Méndez et al. 2019), preventing place-based management of fire regimes and limiting capacity to respond to both new information and changing social and ecological conditions (Platt et al. 2022, Kirschner et al. 2023).

This article feeds into these conversations by outlining a case study where these debates played out in real time. Specifically, this case study focuses on governance developments at the local scale and a situation where such a process led actors to challenge the default policy position that prioritizes broad scale prescribed burning as the best way to reduce the risk of wildfires to communities and the environment. It documents the efforts of a local government in Western Australia (WA) to forge its own unique path toward improved wildfire governance in a place where high biodiversity values coincide with an extreme wildfire hazard rating. The aim of this article is to examine key attributes of a wildfire risk management process to understand how adaptive governance (AG) can emerge in response to specific contextual factors. In doing so, we contribute to discussions about how governance can be more future-oriented by documenting how an emergent decision-making process led this jurisdiction to adopt attributes of an AG approach, effecting positive behavioral change and garnering community support for a new approach to wildfire risk management that is sensitive to local context. The keys to success in this case, along with the challenges encountered, offer useful insights for other governments seeking to determine their own path for governance of linked systems of humans and nature, i.e., social-ecological systems (SESs).

### Adaptive governance and wildfires

AG can take many forms, but it encompasses a family of governance models that integrate principles of resilience into governance, which include anticipating and responding to changes as they arise, as well as developing strategies for dealing with the complexity, uncertainty, and intertwined drivers of change in coupled human and natural systems (Chaffin et al. 2016). Advocates of AG contend that it can improve the fit between social and ecological systems (Folke et al. 2005) and manage the resilience of SESs to natural hazards such as wildfire (Djalante et al. 2011, Abrams et al. 2015, Platt et al. 2022). Resilience is often used to refer to the capacity of a system to recover after a disaster; but a broader conception of resilience that considers adaptation to changing conditions as well as the transformation of SES by changing structures and processes is central to AG (Jozaei et al. 2022).

AG emerged as a strategy in response to the tendency for governance systems to become stuck in “rigidity traps,” with top-down elements inhibiting adaptation, and narrowing down the number of policy and management options that are deemed acceptable (Platt et al. 2022). In wildfire governance, this rigidity could mean a strong preference for prescribed burning or a strong preference for its opposite, fire suppression. For example, in Australia the preference for prescribed burning as a risk mitigation activity has led to the widespread implementation of top-down area-based targets or quotas of hectares burned (Morgan et al. 2020). This preference developed over the course of several decades of intense wildfire events, creating pressure for even more prescribed burning, and ultimately creating inflexible institutional arrangements, organizational inertia, and policy rigidity at higher levels of government (Platt et al. 2022). This has in turn constrained the capacity of local managers to adapt to changing on-ground conditions or make difficult but necessary decisions regarding social and ecological trade-offs (Clement et al. 2016a, Clement 2022).

AG’s calls for flexibility, reflexivity, and policies that anticipate and respond to change mean AG is well suited to wildfires, where much responsibility rests at lower levels and smaller scales. AG seeks to avoid top-down mandates, instead favoring multi-layered governance networks linked across vertical and horizontal scales as a means of mobilizing more resources and fostering innovation. These networks are often polycentric and deliberative in nature, though not prescriptively so (Lebel et al. 2006, Armitage et al. 2012). Consistent with this, moving toward AG generally requires self-organizing networks of local actors, entrepreneurial leadership, and some level of autonomy for environmental managers on the ground (Clement et al. 2016a).

A recent systemic review identified four pillars of governance that require attention to effectively address rigidity traps and confront escalating wildfire risk, i.e., (1) participation of actors in making and implementing decisions; (2) cooperation and co-production among actors within and across levels, scales, and networks; (3) how path dependency and context influences the way actors understand and respond to wildfire risk; and (4) capacity of actors to anticipate and adapt to this risk (Kirschner et al. 2023).

This research touches on each of these pillars by asking: How can dedicating resources (both monetary and nonmonetary) to wildfire risk management planning facilitate characteristics of AG? The case study explores the conditions that might break down rigidity traps, focusing on a local example where actors must address extreme wildfire risk alongside high biodiversity values. The proposition is that participation in this process can support changes to governance that break free of path dependencies by allowing for productive discussions about local context, the status quo and whether it needs to change, and enabling communities and governments to discuss key concerns and trade-offs between valued outcomes at the local scale. However, it also explores the fragility of governance arrangements that emerge through such processes, especially when they hinge on key individuals to maintain momentum.

Empirical case studies where intentional reforms have led to “ideal” forms of AG are scant (Sharma-Wallace et al. 2018), so examining the conditions under which features of AG emerge organically is important for progressing AG in practice. Our case study contributes to this evidence base and offers insight into pathways for governance systems to break out of rigidity taps, and for AG to support adapted forms of policy and risk management options, even as higher level (e.g., state and federal) systems and other attributes of governance are resistant to such change (Clement 2021).

## STUDY AREA BACKGROUND

### Location and development history

**Fig. 1.**
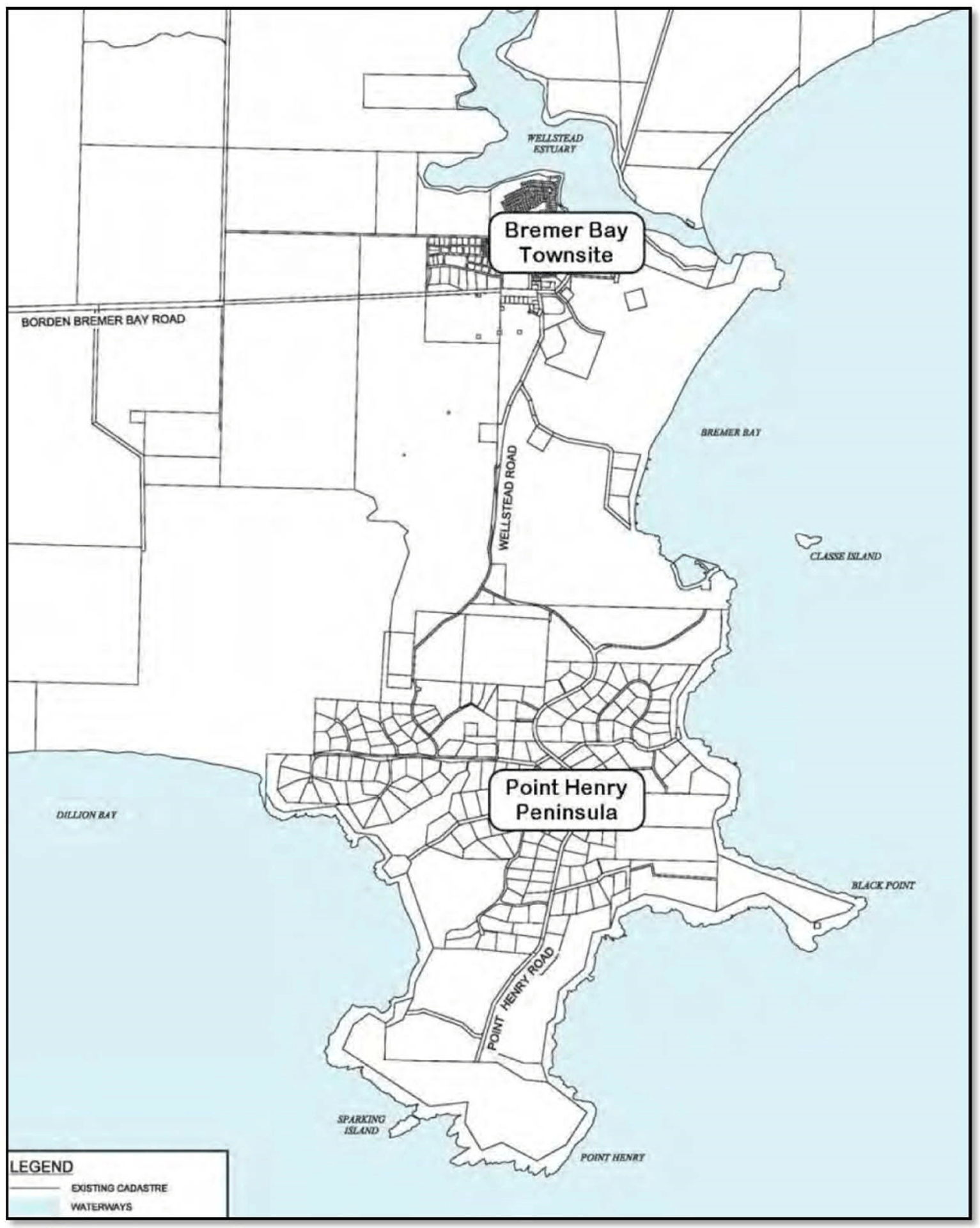
Map of study-area, the Point Henry Peninsula, Australia (TME 2014).

The Point Henry Peninsula (the Peninsula) lies on WA’s south coast within the Great Southern Region, 440 kilometers (km) southeast of the State Capital of Perth (see Fig. 1). The region has a Mediterranean climate, with warm to hot dry summers and mild, wet winters. The Peninsula lies within the Shire of Jerramungup, an economically modest local government area heavily reliant on agriculture that is large geographically (6507 sq. km) but small in population (1133 in 2018). The Peninsula is also part of the Fitzgerald Biosphere, an area known for its high biodiversity values and high levels of endemism, and the boundary adjoins the UNESCO World Heritage listed Fitzgerald River National Park (FRNP).

Formerly a large grazing property, since the 1990s the 2300-ha Peninsula was progressively subdivided to contain 207 freehold properties of 3–10 hectares (TME 2014). Of the 80 properties developed to date, most are zoned “special rural,” with a few larger lots remaining “rural.” Many landowners are not permanent residents. Permanent residents are primarily retirees or/and tourism operators (B&Bs). The remainder of the Peninsula consists of land reserves managed either by the local government or state government agencies.

The Peninsula is generally heavily vegetated and includes mallee shrublands and thickets, woodlands dominated by peppermint and coastal wattle, Melaleuca thickets, and Dryandra dominated mixed heaths (McQuoid and McMahon 2016). The Peninsula’s northern boundary is a 5 km drive from Bremer Bay (pop. 424 in 2021), a small coastal service center and seasonal tourist destination that is the location of the nearest wildfire brigade.

### Fire management background

The Peninsula presents a challenging wildfire response environment. The land use pattern reflects a time when state and local government planning authorities did not prioritize wildfire risk management, and the community was designed to be sympathetic to the environmental and landscape amenities of the Peninsula. Lots are large, only limited clearing of bushland is allowed, and planning criteria ensure that homes and access ways are not visible to neighboring properties.

When the subdivision was initially planned, a decision was made not to require perimeter firebreaks around individual properties. Instead, the subdivision design incorporated a network of strategic firebreaks to provide emergency access points for fire suppression efforts. Some strategic breaks passed through as many as 10 individual properties. Landowners were responsible for maintaining any portion of a strategic firebreak on their property. The piecemeal nature of the responsibility for maintaining the strategic breaks made it difficult for contractors to coordinate gaining access and payments from so many landowners. Over time many segments of the strategic firebreak network became overgrown as maintenance lapsed.

Furthermore, there is no reticulated water supply readily available, and the closest firefighting unit is the volunteer fire brigade in Bremer Bay. Emergency access and evacuation are challenging, with a single roadway providing access to the Peninsula and a subdivision design based on a network of cul-de-sacs. The dominant vegetation is valued for social, cultural, and biodiversity values. Much of this vegetation has high oil content, is very dense, and has not been touched by fire in decades. Because of the nature of the vegetation and rolling topography, the Peninsula has been given an “extreme” wildfire hazard rating (TME 2014).

Several events drew attention to the need for change. In 2002, in the span of only two hours, a wildfire cut a swath across the Peninsula from coast to coast. Although no lives were lost and property damage was minimal, fire crews were trapped in a burnover, making the local fire brigade more aware of the dangers and limited effectiveness of trying to suppress a fast-moving wildfire on the Peninsula. In 2012 a large wildfire in the adjoining FRNP threatened both the Peninsula and Bremer Bay. During that major event, state fire authorities declared the Peninsula as “indefensible,” meaning that because of the high risk to firefighters, authorities would not send emergency response personnel onto the Peninsula until the fire front had passed. A fortunate shift in the weather spared the Peninsula from the fire’s impact but it signaled to the Shire that action was needed. Given the events of the 2012 fire, the increasing number of residents and visitors, and the barriers to effective emergency response, in 2013 the local government identified the Peninsula as its highest priority area for more intensive wildfire risk management.

### Implementing more intensive wildfire risk management and the role of prescribed burning

Governance arrangements for managing wildfires and wildfire risk in Australia involves a complex web of organizations, laws, and policies across local, state, and national levels that cross many different sectors (e.g., environment, land use planning, emergency management, insurance, finance; McCormack et al. 2022). Land management is primarily a state responsibility in Australia, and WA government agencies that bear most of the direct responsibility for wildfire risk management are the Department of Biodiversity, Conservation and Attractions (DBCA) and the Department of Fire and Emergency Services (DFES). Although DBCA is responsible for large-scale burning, this is only on state managed lands. DFES supports local governments to identify at-risk assets and assign treatment options through the development of Bushfire Risk Management Plans, in accordance with guidelines provided by the agency’s Office of Bushfire Risk Management (OBRM 2015). As a local government in a wildfire-prone area, the Shire also must comply with land development controls, including the Bushfire Policy Framework, which is under the purview of the Department of Planning, Lands, and Heritage and includes State Planning Policy 3.7 (Planning in bushfire prone areas), the Planning and Development (Local Planning Schemes) Regulations 2015, and accompanying guidelines. The Shire’s plan (Shire of Jerramungup 2017) lists dozens of national, state, and local pieces of legislation, policies, and guidelines that are relevant to managing risk.

The role that prescribed burning should play in managing fuel loads on the Peninsula has been a divisive topic. In WA, prescribed burning across large areas of landscape is the favored approach to reducing wildfire risk. This preference has sparked controversy, given evidence that the greatest gains are achieved by managing fuels directly around houses and the immediate urban-rural interface (Gibbons et al. 2012, Price et al. 2021); however, these targeted treatments are more costly and socially challenging (Florec et al. 2020). This preference for landscape-scale prescribed burning has been a longstanding practice in WA, with large area-based targets founded on a longitudinal study correlating these practices to reduce area burnt in wildfires between 1953 and 2005 (Boer et al. 2009). These practices have persisted despite increasing evidence that climate change is reducing prescribed burning effectiveness in WA (Campbell et al. 2022) and elsewhere in Australia (Clarke et al. 2022), suggesting a path-dependent rigidity trap that has attracted public scrutiny and debate amongst experts (Clement 2022). Although the targets apply only to state-managed land, this preference for prescribed burning also applies to local governments, including other areas of The Shire, primarily to reduce risk to life and property rather than for biodiversity values, which is another area of debate. Advocates of prescribed burning will often contend that it is necessary because Australia’s ecosystems evolved with fire and some species are fire-dependent. Although this is true, the frequency and intensity of prescribed burning programs can exceed the ability of native flora and fauna to adapt to these altered fire regimes (Gosper et al. 2010, Fontaine et al. 2012) and may even make some landscapes more flammable, especially as the climate change intensifies (Zylstra et al. 2022). The relative importance of different values in fire management is also controversial. Emergency management legislation prioritizes life and property; however, there is a lack of clarity in Australian law and policy about how to consider different values when mitigating risk, particularly in ecologically important areas (McDonald and McCormack 2022).

In this case, some experts and members of the public expressed concerns that periodic prescribed burning of the landscape would diminish the biodiversity and amenity values of the Peninsula. Prior to the establishment of the rural subdivision, a rotational prescribed burning program had been used to support grazing activities on the Peninsula, although grazing to reduce fire risk is a controversial strategy that has not always proven effective (Fensham et al. 2020). Ecological assessments indicated that this had allowed opportunistic native species such as peppermint and wattle to become dominant and weed species to thrive. There were also concerns that prescribed burning could result in an escape and trigger an uncontrollable wildfire event as had occurred in other parts of the state (e.g., 2011 Margaret River wildfire). Still scarred by the 2002 burnover event, and the “indefensible” declaration of 2012, the local wildfire brigade leadership were apprehensive regarding their ability to manage a prescribed burning program in the challenging conditions on the Peninsula (e.g., dense high oil content vegetation in a rolling topography).

### To burn or not to burn

Because of past and current land use patterns the Peninsula is a highly modified environment. This raised an important question: any consideration of trading off some hazard reduction to maintain biodiversity values, needed to be informed by improved understanding of the condition of ecosystems on the Peninsula. Were the remaining biodiversity values sufficient to warrant consideration of such a trade-off?

Competing priorities across complex institutional arrangements create conflicts when considering trade-offs between different values (McLennan and Eburn 2015, McCormack et al. 2022). The effect is similar to that seen elsewhere in the world, limiting flexibility and experimentation with new approaches for managing risk, and defaulting to familiar risk management options (Platt et al. 2022). Against this backdrop of policy rigidity, implementing AG or fostering characteristics that support more locally adapted policies is challenging.

Adding to this complexity within governance, Australia has increasingly embraced the concept of “shared responsibility,” with the idea that it is essential for creating more wildfire resilient communities. What “shared responsibility” means, and its actual effect on resilience, is still contested in law, policy, and practice, because it can also be used by government to devolve responsibility and diffuse blame (Lukasiewicz et al. 2017). However, it has also served to generate valuable discussions around previously hidden tensions between different objectives and values (e.g., amenity, ecological health, and hazard reduction; McLennan and Eburn 2015). Though ambiguous, the concept also has the potential to nicely align with AG principles, which favor de-centralization of both authority and responsibility where possible.

Following the 2012 declaration by fire authorities that the Peninsula was indefensible at the time of the FRNP fire event, the state and local governments hoped that by undertaking risk mitigation measures (including small-scale clearing for fuel management), and creating a safer environment for residents and firefighters, such a declaration could be avoided in future. This hope was dampened early in The Shire’s fire management planning process, when some experts raised concerns that reducing the fuel load to a level that would allow the Peninsula to be defendable in catastrophic fire conditions would necessitate changing the whole heavily vegetated character of the area through some combination of prescribed burning and vegetation clearing. The Shire also received submissions from other stakeholders concerned about the potential impact of such intensive fuel management on the amenity and character of the peninsula.

In 2015, the Shire commissioned an ecological assessment of the Peninsula’s ecosystems to answer that question. The assessment identified 26 native vegetation communities (McQuoid and McMahon 2016). Although past land use practices had allowed opportunistic native species and weed species to thrive, significant biodiversity values still existed and included instances of heathlands classified as threatened ecological communities, i.e., the Proteaceae Dominated Kwongkan Shrubland, which are protected under the federal Environment Protection and Biodiversity Conservation Act 1999 (EPBC Act).

The assessment advocated an adaptive approach to manage both biodiversity values and the wildfire hazard. The approach did not support large-scale prescribed burning or significant vegetation clearing. Instead, it proposed that the composition of native vegetation in disturbed areas be returned to a more natural state through weed management and the selective removal of opportunistic native species. It was also recommended that native woodlands be allowed to reach a climax state rather than aggressively thinning them to reduce their fuel load. The ecologists noted that climax state woodlands in the region tend to be of lower density than less mature woodlands and thus pose a lower wildfire hazard. The network of monitored reference sites established during the assessment would allow the management of biodiversity values to be adjusted as new knowledge was generated.

Based on the assessment, and its engagement with landowners and wildfire experts, the Shire concluded that on balance a prescribed burning program was incompatible with the local risk context. Instead, the elevated fuel loads would be managed through a combination of the following:

1. Limited mechanical treatments (e.g., slashing and mulching) to upgrade the network of strategic firebreaks, widening buffer zones along primary roadways, and creating buffer zones around critical infrastructure (e.g., communication tower);
2. Embracing an adaptive approach recommended by the biodiversity assessment in the hope of both enhancing local biodiversity values and reducing landscape flammability in the longer term;
3. Using planning tools (e.g., Firebreak Notice) and education to encourage landowners to implement wildfire risk mitigation measures on private properties.

This adaptive approach was aspirational in the sense that it had more uncertainty attached to it. There were no obvious examples, at least not in WA, for the Shire to examine to test the viability of the proposed landscape-scale adaptive approach. It would in effect be an experiment that would require many years, even decades, to hopefully achieve its hypothesized benefits.

## METHODS

The case study used data generated for DFES from a research project led by JB (December 2018), focused on the implementation of the Shire’s alternative risk management strategy. The study employed a single-case design using mixed methods. Data sources included: planning and policy documents, site visits, key stakeholder interviews, and a landowner attitudinal survey. Although caution must be taken in generalizing outcomes from single-case studies, they can generate persuasive and rich insights that can help inform management efforts in other contexts that share some of the same characteristics (Yin 2009). Furthermore, contextualizing single-case studies against broader theories, in this case AG, can generate knowledge to extend the practical or operational applicability of those theories (Flyvbjerg 2006). This case has several features that are germane to other wildfire prone areas because it has many of the hallmark features that make change difficult and contentious, including competing values and conflicting mandates that are made even more difficult when multiple levels of governance have responsibility for meeting outcomes.

The study commenced with a review of local government planning documents relevant to the study area and its wildfire risk management history. This included existing local wildfire regulations, wildfire risk management strategies (TME 2014, Shire of Jerramungup 2017), vegetative studies (McQuoid and McMahon 2016), and Shire council meeting notes pertaining to wildfire risk management in the study area.

The next research step included site visits to the study area and meetings with local and regional actors involved in wildfire risk management, including key local government administrators (the Shire CEO and a Shire town planner), the head of the local wildfire brigade, and state government wildfire mitigation officers and wildfire emergency response controllers. Semi-structured interviews with these participants focused on the wildfire risk management challenges of the study area, the successes and failures of past efforts, and their views of the new approach adopted by the Shire to manage wildfire risk in the study area. The site visit included viewing wildfire risk mitigation efforts on the ground and the impact of past wildfires and allowed participants to explain how ecological and social factors interacted with practical considerations for different sites, as well as how and where their efforts required landowner cooperation.

A second site visit focused on interviews with landowners. Interviewing landowners on their properties allowed the interviewer to walk the property with the landowner and have them describe what mitigation measures they had adopted, or were planning to adopt, as well as the challenges at their location (e.g., steep slopes, thick vegetation), and their views of the Shire’s approach.

Of the 21 key stakeholder interviews, 14 were involved in wildfire risk management at the state and local government levels or were non-government actors with relevant expertise in fire management and the remainder (7) were landowners (full and part-time residents). Interviews ranged in duration from 30 minutes to 3 hours. Handwritten notes were taken during the interviews, which were then typed up and subjected to thematic analysis (Braun and Clarke 2006). Stakeholders were identified through a snowballing sample approach, commencing with a seed population of key actors at the state and local levels and using social networks to establish a chain of referrals (Parker et al. 2019). The stakeholder list evolved based on availability and stakeholder recommendations. Care was taken to ensure that the suite of stakeholders interviewed reflected the full range of perspectives on wildfire risk management in the study area. Given the subject matter, the population of potential participants was small, and no new themes or potential participants were identified.

A key theme explored during the interviews was about the emergence of policy networks and their importance for changing wildfire governance, so the interviews explored these social networks qualitatively. Networks, whether organized around particular issues, policies, or communities, are the fundamental building blocks of all governance and empower actors to achieve policy outcomes. As noted earlier, AG draws attention to the importance of self-organized networks in fostering new ideas, and, if they cut across multiple scales, fostering more effective governance. Qualitative social network analysis allows participants to describe, in their own words, the structure and function of networks, and allows for insight into how networks operate in context (Domínguez and Hollstein 2014). The policy networks described in the results are derived from thematic analysis of interview transcripts to describe key individuals and clusters of individuals. This information also provided insights into the informal and formal linkages within actors in the network, structured around shared beliefs and interest in policy making and implementation (Rhodes 2009).

The knowledge gained through the above, along with a review of the behavioral change literature on wildfire risk mitigation, were used to design a landowner survey. The survey sought to obtain landowner perceptions of the level of wildfire risk and their comfort with that perceived risk; gain an understanding of landowner mitigation actions; and assess landowner support for the Shire’s approach to manage wildfire risk.

In July 2018, using the Shire’s landowner database, all 180 Point Henry landowners received a letter seeking their voluntary anonymous participation in the survey. Each received a copy of a questionnaire and a pre-paid return-addressed envelope that landowners could use to return their completed questionnaires. Landowners were also given the option of completing the questionnaire online via a Survey Monkey link. The survey was advertised on the Shire’s Facebook page to encourage participation. After three weeks, landowners received a reminder letter, including another copy of the questionnaire and a prepaid return envelope. The survey was again advertised on the Shire’s Facebook page. In total, 104 questionnaires (59%) were completed with most landowners (83%) choosing to complete the hard copy version of the questionnaire (Appendix 1).

## RESULTS

This research documented the ways that dedicating resources to wildfire risk management planning in collaboration with the community and key stakeholders led to emergence of several elements of AG. Most notably, the process allowed actors to re-consider path dependencies and consider more context-adapted governance. Several conditions supporting this shift are discussed below, i.e., (1) adoption of a systems-based view that cuts across multiple portfolios, (2) emergence of a policy network, and (3) leadership that facilitated an effective planning process.

### Multi-portfolio systems perspective

Following the 2012 FRNP fire event, the Shire felt compelled to take action to reduce the wildfire risk to Peninsula residents and visitors. Participants reported in interviews that initially they viewed the task as a narrowly defined wildfire hazard reduction issue, one that would be limited in scope and resolved within a year or so. There was no explicit consideration of a governance model because the Shire did not anticipate how complex wildfire risk management would become. The initial approach taken by the Shire was consistent with the way that wildfire risk management in WA has traditionally been conceptualized, as a technical risk problem that can be remedied through prescribed burning targets, and walled off from other considerations (e.g., land use planning, biodiversity conservation, differing values).

In interviews, Shire participants also indicated that the Shire had hoped that the declaration of the Peninsula as indefensible during the 2012 fire had created a window of opportunity to get Peninsula landowners to support the Shire’s mitigation efforts and motivate them to act on their own properties. As they started to tackle the problem, it became clear that not all landowners were keen to see aggressive hazard reduction measures implemented on the Peninsula. Shire participants noted that they had realized many landowners were not framing the risk problem in the same way that they were. Many were applying a more holistic frame in which the need to manage hazard levels was part and parcel of a larger view of the Peninsula and its future, especially the values it supports (e.g., amenities and biodiversity). Although the Shire was applying a narrow framing of risk management, others were adopting a systems perspective that crosses multiple portfolios.

The landowner survey provided some additional insights into framing of risk. Like the Shire, landowners believed a high level of wildfire risk existed on the Peninsula. When asked to rate the level of wildfire risk on the Peninsula on a scale from 1 = very low risk, to 5 = very high risk, a large majority rated the risk as either “high” (31%) or “very high” (40%) with only 7% rating the wildfire risk as low (Table 1). However, when asked to rate how “comfortable” they were with the wildfire risk on a scale from 1 = very uncomfortable, to 5 = very comfortable, only a third of landowners were either “uncomfortable” (22%) or “very uncomfortable” (12%) with the level of risk. This means that although most landowners perceived an elevated level of wildfire risk on the Peninsula, unlike the Shire many were comfortable with that level of risk.

The relative weight that should be given to the objective of reducing wildfire risk versus maintaining bushland values became a central issue in the community discourse during the active planning stage and led to some tensions among landowners. This was evident in meetings, interviews, and the landowner survey. When asked whether managing wildfire risk or maintaining ecological values should be given more relative weight in the case of the Peninsula, opinions were mixed (Table 1). Although 43% indicated that managing wildfire risk should be given higher priority, 29% favored maintaining ecological values, and 29% assigned equal importance to the two objectives (Table 1).

Landowner interviews and the open comments to the survey provide additional clues to how some landowners viewed the risk context. Among those comfortable with the existing risk level some viewed elevated wildfire risk as an intrinsic part of the landscape, one they had accepted when they purchased their property: “We knew there was a high risk of wildfire on the Peninsula when we bought the block.” Others expressed fears that wildfire mitigation efforts would go too far (e.g., too much burning or clearing) and diminish the bushland values that attracted them to the area: “The risk of a fire versus the desirability of the location cancel each other out.” Landowners uncomfortable with the perceived level of risk offered a different perspective. They feared that too much emphasis on protecting ecological values would come at a cost to human lives if a significant fire occurred: “Managing the Peninsula like a national park is not a viable solution for residents.”

### The policy network

Interview participants from the Shire noted that they realized the benefit of fostering a network including multiple perspectives to the risk management process. The Shire had limited financial resources, in-house expertise in wildfire risk mitigation, and only one town planner to guide the planning exercise whose time was shared with a neighboring local government. Thus, the Shire needed to leverage its resources and participants stressed the need for community acceptance of its decisions and highlighted that those most impacted by the outcomes of the risk management planning process (primarily Peninsula landholders) had a right, and should be encouraged, to participate. Shire participants noted this acceptance was critical for the legitimacy of The Shire’s decisions and for landowners to undertake appropriate wildfire mitigation measures on their properties.

**Fig. 2.**
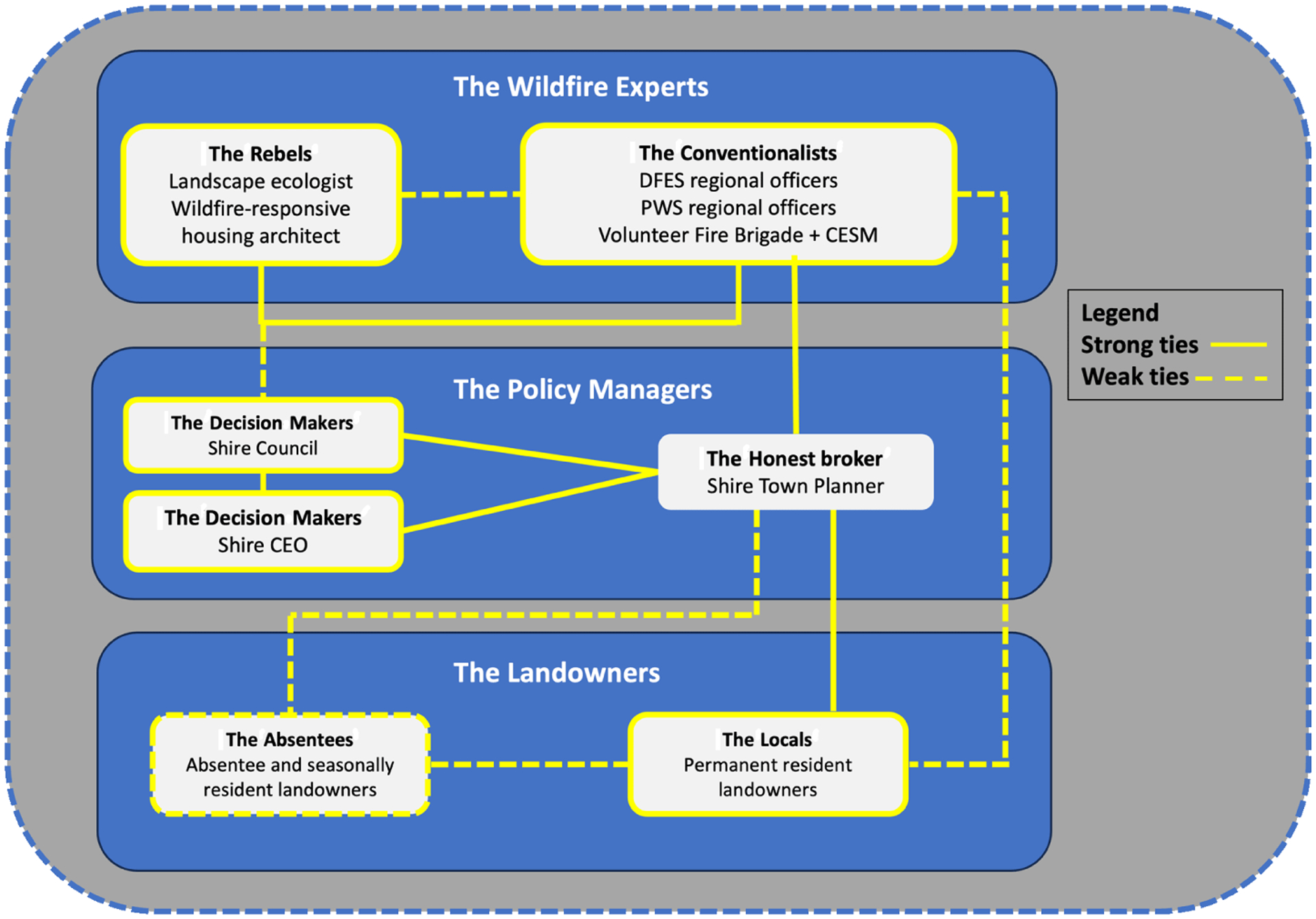
Point Henry Peninsula policy network.

The policy network in the Peninsula case consisted of three clusters of stakeholders: the policy managers, the wildfire experts, and the landowners. Figure 2 illustrates the policy network, clustering actors thematically to illustrate relationships described by participants. The strength of relationships is represented by both strong and weak ties. The strength of ties is a heuristic that represents the extent to which participants described how actors, (i) influence one another; (ii) share similar views; (iii) offer support and help; (iv) communicate effectively regarding complex information and tasks; and (v) are likely to trust one another (Prell et al. 2009).

#### The policy managers

The elected Shire council served as the ultimate decision maker in the policy process. As the only stakeholder elected by the community, the Shire council believed it needed to retain the responsibility and accountability for final policy decisions. This position was not disputed by any of the other stakeholder representatives interviewed and provided the process accountability that is sometimes lacking in self-organized governance networks for ecosystem management (Hahn 2011). The need for public accountability while also allowing for bottom-up self-organization is often considered critical for effectively achieving objectives yet is elusive in examples of AG (but see Green et al. 2016).

Two other key parties in the policy manager cluster were the Shire’s town planner and its chief executive officer (CEO). The Shire’s town planner was an effective policy entrepreneur (Cairney 2018), a role that is often pivotal in fostering new approaches in AG. The town planner drove the policy process on the ground, bridging stakeholder relationships, framing policy challenges, and shaping the deliberative discourse across the policy network. Stakeholders frequently referred to the town planner as an “honest broker” and the central cog in the policy process. The town planner described his approach as pragmatic and noted his belief that both traditional (e.g., prescribed burning) and non-traditional (e.g., allowing vegetation away from property assets to mature and “self-thin”) perspectives on wildfire risk management should be debated as part of the policy process even if that resulted in some tensions among stakeholders. The CEO acted as a conduit between the Shire council as the decision makers and the town planner as the on-the-ground manager of the policy process. The CEO helped ensure that the town planner retained the support of the Shire council, especially at those points in the process when there were tensions within the stakeholder network.

#### The wildfire experts

Like many other rural local governments, the Shire possessed limited within-house wildfire mitigation expertise. On the plus side, the senior leaders of the local volunteer fire brigade were well connected to the state’s wildfire leadership network and had over the years been influential in getting state funding directed to the Shire for wildfire-related activities. However, the expertise of the local fire brigade was in firefighting rather than in implementing risk mitigation treatments. For wildfire mitigation expertise, the Shire relied on its long-standing working relationships with the regional offices of the state’s main wildfire risk management agencies: DFES and the Parks and Wildlife Service (PWS).

At different times within the period 2013–2019, DFES co-funded three contracted wildfire mitigation positions at the Shire. Two wildfire mitigation officers worked with the Shire for two years each. Although these officers did not work exclusively on the Peninsula, much of their effort was spent there because it was the Shire’s highest wildfire risk priority. These wildfire officers conducted on-the-ground hazard assessments, supervised vegetation mitigation activities, and liaised with landowners regarding mitigation measures they could adopt on their properties. Near the end of the policy process DFES funding was used to support a Community Emergency Services Manager (CESM) contract position at the Shire.

There were strong ties among these wildfire experts. For the most part, they shared similar training, were the products of command-and-control style organizations, and had worked together on the ground fighting significant wildfire events within the region. These experts can be described as “traditional” wildfire experts used to working in ways that are different from many of the processes in AG.

There were two other experts who offered counterpoints to the conventionalist expert perspective. One was a full time Peninsula resident who is a consulting ecologist and former FRNP ranger. The other was a seasonally resident architect specializing in wildfire-responsive housing design. They shared a philosophy that wildfire risk management should and can be sympathetic with ecological values. They questioned whether some of the most common mitigation measures such as prescribed burning or significant vegetation clearing were a good ecological fit for the Peninsula. Many of the conventionalists viewed these “rebels” with their unorthodox views as agitators. Shire representatives noted that although these individuals could be frustrating in that they scrutinized their decisions and challenged their way of thinking, the Shire was fortunate to have environmental professionals within their midst, which also lent credibility to efforts to adopt a systems perspective.

#### The landowners

The “landowner” cluster contained two groups: the permanent residents and absentee landowners who resided on the Peninsula seasonally, if at all. Some absentee landowners have dwellings on their properties, while others have yet to develop their lots. The ties between the two landowner groups are weak, as are the internal ties among the absentee landowners. Although they admitted they did not know their absentee neighbors, it was not uncommon for permanent residents to express the view that absentee landowners were not living up to their responsibilities in terms of taking mitigation actions on their properties.

The Shire employed a range of techniques to engage with landowners. There were several community meetings and passive forms of information transfer including letters to landowners, newsletters, notice boards, social media (Shire’s Facebook page), and creating a page on the Shire’s website dedicated to wildfire management on the Peninsula. For two years the Shire hosted a Bushfire Ready group on the Peninsula. Bushfire Ready is a DFES sponsored program that enables local governments to provide a forum for neighbors with the assistance of a trained facilitator to network, share information, and develop strategies to reduce their wildfire risk (Armstrong 2018). As they conducted their hazard assessments and supervised mitigation activities, the Shire’s wildfire mitigation officers provided an on the ground face to the Shire’s efforts. Stakeholders from all three clusters identified the informal encounters between the Shire’s wildfire mitigation officers and landowners as often the most valuable. They provided the opportunity for the officers to explain what risk mitigation actions the Shire was taking, to hear landowner concerns and offer advice on the actions landowners could adopt. They became a valuable conduit between the Shire’s policy managers, wildfire experts, and landowners.

One area where the Shire had only limited success was its engagement with the absentee landowners. Despite efforts tailored to their needs such as holding engagement activities (e.g., Street Meets) when absentee landowners were more likely to be on the Peninsula (e.g., summer school holiday periods), these were met with a tepid response.

### Repurposed planning tools

The Shire was keen to have landowners adopt wildfire risk mitigation practices on their individual properties. Based on their discussions with local stakeholders the Shire did not believe enough landowners would take sufficient mitigation action if a purely voluntary approach was adopted. Instead, creative means were found to repurpose existing planning tools including firebreak notices and licensing agreements to require change on the part of landowners. Using existing policy tools as scaffolding for change in such situations can support transitions to adaptive governance (Clement et al. 2015).

#### Firebreak notice

When the Shire commenced the policy process in 2013, the state government was in the process of drafting wildfire planning reforms (WAPC 2015) that would require that all proposed developments in wildfire prone areas undergo a risk assessment and demonstrate they could meet a set of planning criteria and construction standards, including compliance with Australian Standards 3959 - Construction of Buildings in Wildfire Prone Areas (2009). The Shire wanted to find a mechanism that would result in landowners with existing residences adopting some of the key wildfire mitigation elements required of new residences under the State’s wildfire planning reforms introduced in 2015. In a first for WA, the Shire decided to use its annual firebreak notice issued under Section 33(1) of the Bush Fires Act 1954 to retrospectively introduce mitigation regulation on landowners with existing residences.

In 2015 the Shire revised its firebreak notice to designate the Peninsula as a special area. Developed lots would need to satisfy the following wildfire risk mitigation requirements to be phased in over a 5-year period to disperse the financial burden on landowners:

1. Have a design compliant driveway by Nov. 2016;
2. Include suitable turnaround areas for heavy firefighting vehicles by Nov. 2017;
3. Establishment of a 20,000 liter (L) dedicated fire water supply by Nov. 2018;
4. Vegetation management to create and maintain a 20 m Asset Protection Zone (APZ) by Nov. 2019.

The Shire risk managers were aware of the value of compliance monitoring and had its officers visit all properties to assess compliance progress. Although the Bush Fires Act gave them the power to issue financial penalties for non-compliance, the Shire chose instead to work with landowners to get those properties with shortcomings up to standard. The Shire viewed the issuing of fines as a last resort and likely counterproductive, and at the time of this study no fines had been issued.

#### Licensing agreement

The introduction of a strategic firebreak licensing agreement between individual landowners and the Shire was another innovation. Following engagement with affected landowners on the issue (e.g., community meeting, information on costs and benefits), the Shire introduced a license agreement option. This allowed impacted landowners to voluntarily enter into a deed agreement that granted the Shire the right to maintain and secure access to and use of a firebreak for the purposes of fire prevention and firefighting. The agreement allowed more centralized management of the maintenance of the strategic firebreak network by shifting the responsibility for maintenance away from individual landowners and to the Shire. The licensing agreement is binding on any future owners of the property. Only a couple of landowners have opted not to sign a license agreement. In those cases, the Shire has worked with the landowner to ensure that hazard management occurs.

### Leadership and shared responsibility

The Shire did not want to merely pay lip service to the concept of shared responsibility. For the Shire, achieving a sense of shared responsibility required that all stakeholders, especially Peninsula landowners, were able to contribute to a truly deliberative discourse regarding what a more wildfire resilient (see Clement et al. 2023) future for the Peninsula should look like. Shared responsibility between stakeholders also required that the Shire lead through action, and thus sought to establish a de facto social contract with other Peninsula landowners to collectively take action to manage the wildfire risk. The Shire believed it had a moral obligation to lead through action because it would be requiring behavioral change on the part of Peninsula landowners through the modified firebreak notice. As the various components of the wildfire risk management strategy took shape, the Shire identified several highly visible risk mitigation projects it could easily tackle in the short term. This low hanging fruit took the form of widening the buffer zones along key Peninsula roadways through a vegetation slashing project. In addition to its hazard reduction value, the roadways project signaled to other Peninsula landowners that the Shire was prepared to “walk the talk” and partner with them in sharing responsibility for managing the wildfire risk.

The landowner survey provided an opportunity to test the degree to which Peninsula landowners supported the Shire’s efforts to manage wildfire risk and the extent to which the owners of developed lots were taking actions to mitigate wildfire risk on their properties. When asked how effective the Shire had been in managing the level of wildfire risk on the Peninsula, more than half of respondents indicated the Shire had been either “effective” (49%) or “very effective” (7%; Table 2).

Prior to the survey, the Shire expressed apprehension about how landowners would rate its decision to require mitigation actions via the revised firebreak notice. As it turned out, they need not have been concerned, with strong landowner support for each of the requirements including the most contentious element, the provision of a dedicated fire water supply (Table 3).

Landowners are also holding up their end of the social contract, as they were asked to indicate from a list of 12 mitigation measures those they had implemented (Table 4). The list included three actions required under the revised firebreak notice. Under the revised firebreak notice’s schedule of mitigation actions, landowners should have ensured their driveways and turning areas were compliant by the time of the survey and all landowners indicated this was the case. All landowners also indicated they had made at least some progress toward creating an APZ even though the deadline for compliance was still a year away. Although over half of responding landowners had installed a separate water source for fire suppression, at 56% it was a much lower rate of adoption than that of other firebreak requirements although the deadline for compliance was still several months away at the time of the survey.

If landowners were solely motivated by regulation to adopt mitigation actions, one would expect their actions to be limited to the actions stipulated in the firebreak notice. However, on average survey respondents reported having completed 6.4 actions with 22% having taken 9–10 actions. The dominant voluntary actions were removing combustible materials from around buildings (87%) and sealing gaps to prevent embers igniting the house (72%). The number of actions taken was not significantly correlated with either the level of perceived wildfire risk or the level of comfort with the risk. Landowners may have taken additional actions beyond those regulated out of self-interest or because the policy process had helped create a social norm that encouraged them to take additional action or a combination of both factors.

## DISCUSSION

### Alternatives to landscape-scale prescribed burning

The Shire chose to take the path less travelled and one it would not have predicted when it started the risk management process in 2013. Prescribed burning was not a central part of the strategy, and although expert participants noted the benefits of the proposed approach would take decades to fully realize, leaving significant residual fuel loads in the interim, this aligned with AG recommendations to think across longer time frames.

Based on community deliberations, the Shire believed this combination of actions best threaded the needle in the desire to reduce the wildfire risk on the Peninsula while maintaining the natural bushland amenity and biodiversity values that people valued. Its risk managers described the strategy as having pragmatic and aspirational elements. Using mechanical treatments in high priority locations would allow some much-needed reduction in fuel loads. Albeit much less than a prescribed burning program would offer, but without the risk of an unintended wildfire event triggered by a prescribed burning escape and greater attention to fire regimes that are potentially more appropriate for local ecological conditions.

When the Shire came to understand the diversity of views among the Peninsula community and realized that technical questions about how to reduce hazards could not be separated from larger value-laden questions, it adjusted its risk management process. The Shire realized the need to further engage stakeholders in the risk management process, as not allowing stakeholders the time and means to deliberate options would likely only result in community rejection of any Shire decisions. Instead, the Shire chose to embrace the role of facilitator of a discourse among Peninsula landowners and wildfire experts about how to balance wildfire risk management objectives with other planning objectives,; the most notable being maintaining landscape amenity and biodiversity values. The Peninsula would be viewed as an SES in which humans are part of rather than separate from nature. The Shire also made a concerted effort to reduce any preconceived notions it had of preferred solutions and embrace the uncertainty associated with considering a full range of orthodox and unorthodox perspectives and potential solutions.

Interestingly, about two years after the Shire had shifted course to embrace a more holistic and multi-portfolio approach to its wildfire risk management process for the Peninsula, it joined the state government’s newly created wildfire risk management program for local governments. Driven by guidelines established by WA’s lead fire authority, DFES, the program provided a step-by-step expert-driven methodology to technically assess the wildfire risk to individual assets (e.g., homes; OBRM 2015), set priorities, and select mitigation measures. Local governments that produced a satisfactory wildfire risk management plan qualified to apply for mitigation funding from the state government. State policies and guidance tend to focus on both social and ecological values but give primacy to the goal of reducing risk to life and property. One of the Shire’s innovations was to approach risk management in a more holistic way, which is a small but important shift from the status quo, based on discussions about what the community valued.

### Lessons about AG

Although AG can be an intentional strategy (Brunner 2010, Clement et al. 2015), it can also emerge in cases as an adaptation to challenging conditions, particularly when windows of opportunity arise (Folke et al. 2005). The Peninsula case is an example of the latter, where several key features of AG emerged as a by-product of efforts to foster resilience to wildfire. Although the ultimate outcome of these efforts in terms of wildfire risk may not be known for several decades, there are still several lessons to be learned from this case, both about conditions for success and the challenges that face not just Point Henry, but many communities around the world facing extreme wildfire risk.

Adopting a systems-based, locally adapted approach that cuts across multiple policy portfolios was a key AG feature that emerged in this study. This is notable in part because the narrow framing of risk management typically adopted by policy makers in Australia undermines the holistic approach needed to deal with the risks of wildfires alongside other policy imperatives (e.g., biodiversity conservation, providing for amenity and tourism values; Ruane 2020). It is also notable because wildfire risk management is a policy arena that is known to be separated into silos and struggles to provide pathways for understanding how communities might “live with fire” (Paveglio et al. 2018). Often the focus of AG reform is on shifting perspectives on decision making, but this case demonstrates the important role of how communities frame both problems and solutions. Such frames influence how problems are defined, who and what is considered relevant to causing and resolving those problems, what solutions are favored or discounted, and thus shape the outcomes of policy interventions (Clement 2021).

Although stakeholder engagement is of critical importance to AG, this does not always guarantee a change to the status quo or move to options that might better foster resilience for adaptation currently or transformation in the future (Jozaei et al. 2022). In this case there was already alignment because the community held a more holistic perspective on how wildfire management might sit with other values they held for the peninsula. Large-scale prescribed burning is the favored public policy response to wildfire risk with the view that it is essential to help the public “feel safe” (Clement et al. 2016b). Community support for large-scale burning to protect life and property is often invoked as a reason to maintain current practices, but this is not supported by robust evidence and researchers have called for meaningful engagement with communities to consider values and trade-offs (Clement 2022, McDonald and McCormack 2022). The survey results suggest that they were not in denial of the risks they faced, but rather cognizant of the fact that there are trade-offs between risk and reward (i.e., amenity and biodiversity values). Those results also suggested motivations beyond just “rules-on-paper” (i.e., regulations), suggesting that community norms also helped facilitate the transition. Community involvement is critical to AG, and yet it is often an underappreciated element of successful examples where AG has emerged (Green et al. 2016). Here it seems the ways in which the community framed both risk and “success” helped to form an important social contract that underpinned the shared approach to fire management.

The Shire’s genuine interest in deliberation and the notion of shared responsibility being a “two-way street” is also a factor in the transition to AG. Although the AG literature focuses strongly on participation and co-production with local communities and other stakeholders (pillars 1 and 2 in Kirschner et al. 2023), most governance models still adopt fairly standard models of consultation, focusing on those elements required by law or for political support. Rather than having a particular view on what should be done and seeking support from the community, in this case, the Shire demonstrated genuine openness to what the community might want. The results of this study provide insights into how co-production can foster innovation and affect both risk perceptions and behaviors, which ultimately could improve outcomes for communities. However, this case study also highlights the fragility of relying on a few key actors and a time- and resource-limited planning process, as progress stalled once those elements were removed. This underscores the importance of “institutional work,” which refers to the strategies that actors use to change the cognitive, normative, and regulatory elements of institutions. Different strategies are required to innovate (developing new institutions), disrupt (challenge current institutions), and maintain (uphold those new institutions; Lawrence et al. 2009). This case study reinforces the importance of attending to this third category, which requires active work to valorize and routinize changes that emerge, while also narratives about why these changes are an improvement for people in the environment. This allows changes to be embedded in how actors think and make decisions, which is critical for achieving lasting change in wildfire governance (Clement 2021).

The case also demonstrated the importance of policy networks at the local level for broadening the response to wildfire risk, enabling consideration of strategies beyond the traditional prescribed burning remedies. The need for diversity and redundancy is a common recommendation in the AG literature to enhance SES resilience (c.f. Chaffin et al. 2016). Diversity requires overcoming path dependency, and in this case, strong ties between key actors within this policy network (Fig. 2) allowed local actors to partially overcome the strong pressures toward “business as usual” among the state actors in this network. The idea that some ecosystems require longer burning intervals (c.f. Zylstra et al. 2022) is controversial in Australia because it is antithetical to the “fighting fire with fire” approach that prevails in the country and particularly in the state. This differs from the focus on suppression in biodiverse areas in the United States (Platt et al. 2022), but the same principle applies when escaping rigidity traps. This study shows that the public is capable not only of considering other approaches but also of understanding the extent to which risks can be managed and accepting residual risks in fire-prone areas. Difficulties maintaining the changes once the network changed, however, suggests there was a lack of redundancy.

### Challenges

Despite having mapped a pathway to a more wildfire resilient future on the Peninsula, the outcomes remain fragile because of several uncertainties. AG is an approach that calls upon decision makers and stakeholders to continually adapt and improve, and this case raises important questions about how to not only support such growth, but also just maintain. There are several factors that will make it challenging to ensure positive changes continue, and those factors are often outside of the Shire’s control. Important factors for governance of SES such as monitoring, choosing context-based management interventions, communication and information sharing, and leadership is only partially within the control of local governments. Even where AG features emerge locally, they may fail to lead to more effective governance if they are not maintained. This study draws attention to the importance of redundancy and dedicated institutional work not just to overcome path dependency, but also to embed and maintain changes and build capacity across scales to better govern resilience of SESs (pillars 3 and 4 in Kirschner et al. 2023).

There are also outstanding questions about whether this apparent shift toward AG will be maintained and whether the Shire will be able to continue to adapt over the longer term. An interesting feature of this case study is how resources, structures, and human agency interacted to facilitate some features of AG, but how this may not prove sustainable over the long term. These three interconnected factors are considered critical for building adaptive capacity and fostering resilience (Clement 2021). In Point Henry, both tangible aspects (e.g., finances for wildfire planning and staff) and less tangible aspects (e.g., entrepreneurial leadership, community values) aligned to create an opportunity for human agency to play a bigger role.

For example, the AG literature has a strong focus on the critical role of entrepreneurial, structural, and intellectual leadership in supporting effective action that is adapted to local conditions (Olsson et al. 2006, Rijke et al. 2012, Clement et al. 2016b). This case confirms that importance but also highlights how precarious that can be. Several key individuals played outsized roles in driving the policy process to a successful outcome. A resource poor local government may struggle to hold on to or successfully replace such in-demand talents in the longer term. The adaptive approach to vegetation management on the Peninsula will require many years, even decades, before the full benefits can be realized. Although the current Shire council has been willing to support such a non-traditional approach, it is not clear that future councils will have a similar view or be willing to support an experimental approach rather than reverting to a more orthodox hazard reduction strategy such as prescribed burning.

In this case, access to resources and institutional structures enabled actors to consider how they wanted to respond to local conditions that make them particularly vulnerable to wildfire and act deliberately on this. This window of opportunity was very limited, however, with dedicated resources for risk management planning in the Peninsula provided for only 1–2 years. Without resources and supportive structures at the state and federal levels, it may undermine longer term resilience. The Shire’s reliance on short-term state government grants to support its mitigation projects and fund in-house wildfire management officer positions draws attention to what seems to be an eternal battle between problems that require recurrent funding, and the prevalence of models for specific projects (Clement et al. 2016a). As more local governments undertake wildfire risk management planning, it is uncertain if the state’s mitigation funding pot will be proportionately expanded or if the Shire will continue to be successful in winning grants. A lesson from this case on overcoming rigidity traps is that self-organizing policy networks and leadership can be critical in shaping participation and co-production (pillars 1 and 2), helping to overcome path dependency (pillar 3); but for those changes to be embedded, dedicated resources (monetary and nonmonetary) are needed to build capacity over the longer term (pillar 4 in Kirschner et al. 2023).

## CONCLUSION

Although this is a promising case study, it is still a departure from the ideal forms of AG that are posited to lead to better outcomes in wildfire governance (Clement 2022). Governance reform is difficult to intentionally guide. In this case, emergent AG properties were the result of a planning process that sought to improve the fit of wildfire risk management and the local SES. Acknowledging the challenges just outlined, some keys to success can be identified in the emergent features of this case study.

### Keys to success

The approach to governing wildfire risk in this case was quite unlike the standard approach in WA in several ways, embracing both a unique approach to managing (and accepting) risk, and features that more closely resemble AG. The keys to success in this case provide insight for overcoming rigidity traps:

1. When the Shire realized that the risk problem was as much about value choices as it was technical hazard assessment, it changed its wildfire risk management process to facilitate a community-wide deliberation of what becoming a more wildfire-resilient Peninsula would involve.
2. Although it was the ultimate decision maker, the Shire made a concerted effort not to pre-empt the outcomes of the deliberative process and to allow all perspectives to be aired even if they were outside the orthodoxy of wildfire risk management.
3. The Shire successfully leveraged its limited financial and technical resources through its strong working relationships with the state’s key wildfire management agencies.
4. The person managing the day-to-day administration of the Shire’s risk management process was viewed as a respected honest broker by all stakeholders.
5. When knowledge gaps emerged during the deliberative process the Shire took action to fill them such as commissioning the biodiversity values assessment.
6. The Shire made the concept of shared responsibility more than a slogan. By undertaking highly visible projects, they signaled to landowners that the Shire would be partnering with them in forging a wildfire-resilient future on the Peninsula.
7. The Shire did not rush the risk management process and gave stakeholders the time needed to grapple with challenging value-laden issues, absorb new information, and arrive at informed judgments.

What remains to be seen is whether AG will foster resilience to wildfires and whether this is a net benefit to the community or the ecosystems on the peninsula. These are still open questions, and ones that may not be answered for years to come. Although the Shire did not set out to employ an AG approach to its wildfire risk management, the policy process that evolved reflects many of the attributes of AG. The policy process transitioned from one that initially had a narrow hazard management focus to an integrated planning approach involving multiple policy portfolios (i.e., land use planning, hazard reduction, biodiversity conservation). It allowed innovative local solutions to emerge from robust deliberation within a network of local stakeholders regarding how best to manage wildfire risk on the Peninsula. It created a safe public space for stakeholders to debate challenging topics such as how much risk is too much and what might a more wildfire-resilient future look like. It encouraged a sense of shared responsibility that saw both the Shire and other landowners implementing wildfire governance measures. The sustainability of the outcomes of the wildfire risk management process remains a question with the insecurity of political, financial, and human resources the most notable. Despite this, the many positive outcomes of this case study should encourage other local governments around the world to consider adopting an AG approach as they seek to determine their own pathways to more wildfire-resilient futures for at-risk communities.

## Figures and Tables

**Table 1.** Landowner perceptions of risk and comfort (n = 104), and importance of maintaining ecological values versus managing wildfire risk (n = 101).

Level of risk (n = 104)	Percent	Level of comfort (n = 103)	Percent
Very low risk	0	Very uncomfortable	11.65
Low risk	6.73	Uncomfortable	22.33
Moderate risk	22.12	Neither	16.50
High risk	30.77	Comfortable	42.72
Very high risk	40.38	Very comfortable	5.83
No opinion	0	No opinion	0.97
	100.0		100.0
Response categories		Frequency	Percent	
Maintaining ecological values is much more important		11	10.89	
Maintaining ecological values is somewhat more important		18	17.82	
Equal importance		29	28.71	
Managing wildfire risk is somewhat more important		25	24.75	
Managing wildfire risk is much more important		18	17.82	
		101	100.00	

**Table 2.** Shire effectiveness in managing wildfire risk (n = 98).

Response categories	Percent
1. Very ineffective	5.10
2. Ineffective	13.27
3. Neither effective / ineffective	17.35
4. Effective	48.98
5. Very effective	7.14
6. No opinion	8.16
Total	100

**Table 3.** Landowner support for revised fire break notice requirements.

Level of support / opposition	Fire vehicle turnaround	Driveway	Dedicated fire water	APZ^†^
	% (n = 102)	% (n = 102)	% (n = 101)	% (n = 102)
Strongly oppose	6.86	7.84	13.86	16.67
Somewhat oppose	7.84	6.86	11.88	8.82
Neutral	9.80	10.78	17.82	7.84
Somewhat support	19.61	24.51	12.87	19.61
Strongly support	52.94	48.04	41.58	45.10
No opinion	2.94	1.96	1.98	1.96
Total	100	100	100	100

† APZ = Asset Protection Zone.

**Table 4.** Mitigation actions taken by landowners with developed lots (n = 54).

Mitigation actions	Freq.	%
Firebreak notice requirements		
(a) Improved access/egress and turning areas for fire vehicles	54	100.0
(b) Reduced vegetation around house/buildings to create a safety zone (e.g., Asset Protection Zone)	54	100.0
(c) Installed a separate fire suppression water source	30	55.6
Other mitigation actions		
(d) Removed combustible material away from the house (e.g., gutters)	47	87.0
(e) Sealed gaps and spaces to prevent embers igniting the house	39	72.2
(f) Obtained suitable hoses or equipment to defend my property in a fire	31	57.4
(g) Installed an independent power source for water pumps	30	55.6
(h) Planted fire resistant plant species near sensitive assets	15	27.8
(i) Installed a sprinkler system on the house or other buildings	14	25.9
(j) Installed a reticulation system to wet vegetation in case of a fire	13	24.1
(k) Created a refuge of last resort on my property	12	22.2
(l) Installed double glazing of windows	5	9.3
(m) No action has been taken on my property	0	0.0

## Data Availability

Data can be made available upon request to the Department of Fire and Emergency Services. Ethics and data protection guidelines limit publication of the full dataset.

## Footnotes

[1] In Australia the term “bushfire” is preferred over the term wildfire, but here we use wildfire throughout because it is the more common term internationally.
